# Developing E-cigarette friendly smoking cessation services in England: staff perspectives

**DOI:** 10.1186/s12954-018-0244-8

**Published:** 2018-08-03

**Authors:** Hannah Farrimond, Charles Abraham

**Affiliations:** 1 EGENIS (Exeter Centre for the Study of Life Sciences), Department of Sociology, Philosophy and Anthropology, University of Exeter, FF16, Byrne House, Streatham Campus, Exeter, EX4 4PJ UK; 2 School of Psychological Sciences, Faculty of Medicine, Dentistry and Health Sciences, University of Melbourne, Parkville, Victoria, 3010 Australia

**Keywords:** Smoking cessation, Stop smoking services, Qualitative, Harm reduction, E-cigarettes, Vaping, Tobacco control

## Abstract

**Background:**

Public health leadership in England has taken a distinctive international stance by identifying the potential public health benefit of e-cigarettes for smoking cessation. This includes the development of a ground-breaking set of national guidelines for developing e-cigarette friendly stop smoking services. However, little is known about the views of staff engaged within these services and whether or how such services are becoming e-cigarette friendly. This study aimed to investigate the uptake and usage of e-cigarette guidance, from the perspective of those enacting tobacco cessation interventions ‘on the ground’.

**Methods:**

Qualitative semi-structured interviews were conducted with 25 cessation service staff, including advisors (*n* = 15), managers (*n* = 5) and commissioners (n = 5) from eight different services in the South-West of England, UK. A thematic analysis of the transcripts was conducted using NVivo software.

**Results:**

Although some stop smoking services labelled themselves e-cigarette friendly, there was no consensus over what this should entail. For some, this meant active engagement, such as working with local vape shops, and in the case of one service, offering e-cigarettes through a voucher scheme to disadvantaged groups. For others, an e-cigarette friendly service was conceptualized in a passive sense, as one which welcomed service users using e-cigarettes. Many services did not use the ‘e-cigarette friendly’ claim in their branding or promotional material. Several discursive themes underlay differing staff attitudes. Those more reluctant to engage framed this in terms of their ‘duty of care’, with concerns focusing on the addictiveness of nicotine, lack of medically licensed product and ongoing scientific controversy. Those motivated to engage drew on a discourse of social justice goals and *‘*doing things differently’ in relation to lower socio-economic status smokers, those with mental health issues and other vulnerable groups. Strong public health leadership was also identified as a key factor in changing staff attitudes towards e-cigarettes.

**Conclusions:**

On-the-ground enactment of e-cigarette friendly services is varied as well as reflective of the wider policy and regulatory environment. Although the context of English stop smoking services is one of austerity and change, there are opportunities for active engagement with e-cigarettes to achieve overall cessation goals. For this to occur, training, policy consistency and sharing best practice are needed.

## Background

E-cigarettes deliver nicotine through vapour rather than combustible means as with tobacco smoking.^1^ There is a lack of international consensus over the public health role for e-cigarettes [1]. Debate has focused on the relative estimates of the health benefits of vaping compared with tobacco smoking [2, 3], the unknown long-term risks [4–6], the role of flavours [7, 8], their use by minors/children [9], their effect on bystanders [10, 11] and their effectiveness for tobacco cessation [12–15]. Using e-cigarettes as a tool for smoking cessation within health-care services is controversial. Proponents of e-cigarette use, such as Public Health England (PHE) and NHS Health Scotland, have suggested vaping may have a role to play similar to existing therapeutic products, such as nicotine replacement therapy (NRT) varenicline and buproprion [16–18]. In contrast, public health bodies such as the World Health Organization have been more cautious [19, 20].

Given this lack of consensus, regulatory regimes have become divergent [21]. In countries such as Singapore, Thailand, India and Australia there are tight regulatory regimes that either ban or heavily restrict accessibility to e-cigarettes (e.g. [10, 22]). Others, such as Canada and New Zealand, have moved recently towards legalizing and regulating vaping [23]. To some extent, this can be characterized as an ideologically driven debate between abstinence advocacy and harm reduction approaches to drug use, with the latter favouring incorporation of e-cigarettes into smoking cessation services [24, 25] (although see also [26]). Divergence also reflects the paucity of conclusive evidence alongside the lack of existing market regulation for such products. In Europe, the Tobacco Products Directive (TPD) of 2016 makes provision for medical licensing of products as part of a twin-track approach, alongside consumer regulation. However, no such product is currently on the market and licensed [27].

Guidance for English cessation services to become e-cigarette friendly is ground-breaking and certainly in opposition to some international policy positions. The rationale for including e-cigarettes is to combine the most popular method of quitting [28] with the most effective; behavioural support plus pharmacotherapy [29]. According to guidance produced by the National Centre for Smoking Cessation and Training (NCSCT) in 2016, an e-cigarette friendly stop smoking service is defined as one who ‘supports clients who want to use an e-cigarette to help them quit smoking and reaches out to smokers considering using an e-cigarette to come to the service for behavioural support’ ([30], p. 10). Advisors are recommended to familiarize themselves with e-cigarettes (e.g. by reading forums, visiting a shop) and be positive in their language (e.g. do not say ‘we can’t recommend one’ which might sound condemnatory, rather say ‘we can’t supply them, but we can certainly offer the extra support…’) (p. 10). The guidance also suggests that services do not challenge long-term e-cigarette use as it may be protective against relapse (p. 10).

The NCSCT report is part of a complex picture of guidance about using e-cigarettes for smoking cessation which has emerged in the past 5 years in the UK. Public Health England released a seminal report in 2015, updated in 2018, stating that e-cigarettes are approximately 95% safer than tobacco cigarettes [16, 27]. Action on Smoking and Health (ASH) (a campaigning charity influential in tobacco control) supported this stance with their 2014 and 2016 briefings [28, 31] as did the Royal College of Physicians [32]. In contrast, in 2016, Public Health Wales were considering a legislative ban on e-cigarettes in some public places. More recently, in 2017/8, policy statements from UK Public Health bodies have aligned in relation to encouraging e-cigarette users to use stop smoking services [18, 27, 33]. National Institute for Health and Care  Excellence (NICE)’s guidance is slightly more cautious. It recommends health professionals give information about e-cigarettes but does not list them as evidence-based interventions for stop smoking services [34]. In comparison with international examples, the policy guidance to incorporate e-cigarettes into cessation services in England and now in the wider UK is distinctively positive. Little is known, however, about if and how staff in such services have responded to the call to be e-cigarette friendly.

The English stop smoking services, which are free at the point of use, are almost unique internationally. Their long-term effectiveness has been tracked over time [35] and the basis for their success theoretically articulated [36, 37] and empirically evaluated [38]. However, recently, there have been multiple changes to these services. First, they have been moved from National Health Service management to local authority control. Funding has been cut by 50% [39] and 25% of local authorities no longer commission specialist stop smoking services [39]. Second, there has been a transition towards integrating smoking cessation into other ‘lifestyle’ or ‘wellbeing’ services [40]. Third, there has been a focused targeting of vulnerable/‘hard to reach’ smokers (e.g. who are lower socio-economic status, have mental health issues and/or co-occurring addictions, also pregnant women) to close the health inequalities gap [41]. These groups can be harder to attract, retain and treat successfully within services [42]. Finally, there has been an overall decline in use of cessation services. The introduction of e-cigarettes is understood by staff to be a key reason for the drop in footfall [43]. Thus, the decision to consider e-cigarette use as part of cessation services may not only be ideological but pragmatic: ignoring e-cigarettes could render stretched services obsolete. Other staff may fear e-cigarettes are hastening their decline.

International research has begun to delineate health professionals’ perspectives on e-cigarettes. For example, a recent study of doctors and tobacco counsellors in the Netherlands found that although a majority saw vaping as less risky than tobacco smoking, they did not see it as an effective cessation aid and did not strongly recommended it to their own patients [44]. Research from the US on professional attitudes has found them predominantly negative toward e-cigarettes due to lack of evidential certainty, leading GPs [45] and quit line professionals [46] to reject recommending them. However, there are signs that this dominant negative response may be changing. Recent research with US patients who were smokers found that over half of their personal physicians had spoken to them about e-cigarettes as potential quit aids [47]. Similarly, a qualitative study of US physician experiences with e-cigarettes found that although physicians were ambivalent about recommending e-cigarettes due to uncertainty over long-term effects and safety, they were not averse to doing so, particularly if new/more positive evidence became available [48]. A US study of junior doctors found that those who recommended vaping therapeutically were more likely to hold a harm reduction model of treatment rather than an abstinence one [49]. Overall then, a heterogeneous picture is emerging, where negative attitudes are the norm, but also where shifts over time, and shifts in thinking by health care professionals are also occurring.

In England, Hiscock and colleagues have tracked changes in stop smoking service staff attitudes since 2011 through a series of surveys. Practitioners reported both greater interest in and use of vaping amongst clients at later time points [43, 50]. Practitioners themselves have also become positive about e-cigarettes over time, with 15% agreeing or strongly agreeing ‘e-cigarettes are a good thing’ in 2011, to 26% in 2013, and 24.4% in 2014. Of course, this still indicates that the large majority of staff do not see e-cigarettes as a good thing. Furthermore, differences emerged between professional roles, with those with a more policy-oriented role, such as managers and commissioners, being more positive about e-cigarettes than those working directly with smokers [43]. In line with the international research, practitioners were concerned about the addictiveness of e-cigarettes, and their safety/effectiveness, as well as the lack of licensed product [50, 51]. One qualitative study, which examined both stop smoking user and advisor attitudes, found that uncertainty was foundational to ambivalent attitudes towards e-cigarettes [52]. From the perspective of clients using cessation services, the safety of e-cigarettes is their primary concern [53, 54]. Furthermore, some clients have reported other people’s long-term use of nicotine via e-cigarettes could be potentially threatening to their own nicotine abstinence goals [55].

Innovative practice is emerging. One pilot funded initiative in London has incorporated e-cigarettes as part of its cessation treatment [56]. They found clients to be positive about their use, with particular success when combining e-cigarettes with varenicline [56]. Other stop smoking services, such as Leicester City, Bristol City and Hampshire (Quit 4 Life), have reported trialling the provision of e-cigarettes or vouchers to clients, but no research is currently published.

The existing research on stop smoking staff attitudes was conducted prior to the publication of the Public Health England report (2015) and the NCSCT guidance (2016). This study therefore investigates how these macro-level national policy interventions have or have not shifted attitudes and practices with cessation services in what is a fast-moving context. Studying staff beliefs and experiences in relation to e-cigarette friendly services is important for several reasons. First, it offers an opportunity to ascertain how policies or training guidance are being interpreted in practice. Lipsky has argued that front-line staff are the site of policy enactment in public service, and it is their ‘discretionary’ application of top-down edicts that determines whether or not changes occur in day-to-day practice [57]. Second, it allows the qualitative exploration of both ideological and practical challenges that e-cigarettes might pose within professional practice. Staff are schooled within a medical model of smoking cessation including pharmaceutical treatment for nicotine dependence [58]. E-cigarettes are a consumer-led technology which has originated outside the medical sphere [59]. This may create problems for service integration.

This study sought to investigate these issues through a qualitative exploration of how cessation staff experience the challenge to be e-cigarette friendly within their services, given wider national and international policy contexts.

## Method

### Design, sample and procedure

The data reported here was drawn from a larger qualitative study investigating stop smoking services responses to e-cigarettes, including observational and interview data with staff, clients and users of vape shops. A purposive sampling strategy was used [60], aiming to ensure a spread of experience and staff roles. Qualitative semi-structured interviews were conducted with 25 cessation services/tobacco control staff, in the following broad categories: advisors (*n* = 15), managers (*n* = 5) and commissioners (n = 5), although two ‘commissioners’ also had managerial roles in services because the supply/commissioning division had been abolished. Advisors saw clients on a daily basis delivering cessation interventions face-to-face and by telephone, also running groups/outreach in the community (e.g. with pregnant women, with local addiction groups). Managers/leads had a role in overseeing cessation advisors and community staff (e.g. pharmacists) and in devising local tobacco control strategy in line with their contracts and national policy. Commissioners were responsible for issuing tobacco control contracts alongside other public health spheres as well as devising/overseeing policy initiatives.

Initial recruitment was conducted through the Public Health England (PHE) Tobacco Control network in the South-West which is a local network organized by PHE to disseminate policy and share best practice, involving all tobacco managers/commissioners in the region. The first author gave a short presentation about the project at a quarterly meeting. Managers/commissioners who expressed interest at this meeting were then approached formally by email with written information. Managers further disseminated the invitation to participate to their advisors. This author also visited two services to explain what participation would involve to the whole team. Out of 11 services in the South-West network, 8 had at least one member of staff participate, ensuring a spread of services were represented.

Interviews were conducted primarily at the service workplace, face-to-face, in separate rooms for privacy reasons (*n* = 21). Some phone interviews of managers/commissioners were also conducted (*n* = 4). Recruitment was stopped when saturation of experience/themes was reached.

Data was collected from December 2016 to March 2018. The data collection timespan was relatively long because services were undergoing restructuring/change during this period.

The relevant NHS and university ethics committee approved the study. Participants signed and returned an information/consent sheet, retaining one copy for themselves, consenting to the recording, transcription and use of their interview for academic purposes. In the text, participants (indicated by P below) are anonymized and denoted by their role (e.g. advisor, manager, commissioner). Because of the changes to services, and their divergent structures, job titles were often very distinct (e.g. health improvement officer, lifestyle advisor). These were altered in the text to ensure anonymity and to make their role clear.

### Measures

Interviews were semi-structured in format, based on an interview schedule [60]. Initial wide-ranging pilot interviews with one manager and one advisor were conducted and the final schedule developed from these. Interview questions covered (a) the person’s job role; (b) the structure of services and recent changes; (c) whether services used the phrase ‘e-cigarette friendly’ and if so what this meant, including any interaction with vapers/vape shops; (d) personal experiences and opinions on e-cigarettes within services; (e) policy and media issues with e-cigarettes (e.g. changes in risk perceptions, scientific evidence, media stories) and (f) how they saw the future unfolding in relation to e-cigarettes.

### Analysis

Transcripts were coded using NVivo software, using Braun and Clarke’s method [61]. There are two stages to this (a) initial descriptive content codes are generated then and (b) these are drawn together in ‘higher order’ analytic themes to produce an interpretation. This is both a top down and bottom up process. Themes were generated from the material itself (e.g. on recent changes to services) as well as from previous reading/literature (e.g. health professionals’ uncertainty about the scientific evidence). The first draft of the analysis was generated by the first author and reviewed/reworked by the second until interpretation was agreed.

## Results

The findings are structured into three major themes: theme one concerns the activities and attitudes of services in relation to becoming e-cigarette friendly, including their underlying values; theme two locates staff-identified barriers to integrating e-cigarettes into services, and; theme three analyses the role of public health leadership and guidance in driving e-cigarette friendly services.

### Theme one: active and passive approaches to being e-cigarette friendly

#### The changing context of stop smoking services

The interviews revealed that staff were working in the context of considerable change, both in their own roles and in the wider structures of local public health: ‘we’ve been through *a lot* of change’ (P49, manager). Many staff now had a remit to work on lifestyle change in the wider sense, including weight loss, not just tobacco control. Job titles were also changing to reflect this, such as ‘wellbeing practitioner’ or ‘health improvement advisor’. There was also a renewed emphasis on targeting ‘hard to reach’ or ‘disadvantaged’ smokers with the most intensive behavioural support (e.g. face-to-face counselling):

There’s pockets of deprivation across the city, quite extreme health inequalities, and so we target, we run our clinics in key deprived neighbourhoods…we do some work with the mental health community outreach teams…although [those smokers] are incredibly difficult to engage with (P49, manager)

In some services, this was coupled with a withdrawal of services from clients deemed to require less intervention (e.g. offering them online or telephone services). Most services had also seen cuts to staff numbers or to health promotion provision. The need to consider e-cigarettes as part of treatment protocols was therefore part of a wider context of change within stop smoking services:

We know that we were not seeing the numbers…and that’s when e-cigs came in, we were already having less people coming through (P46, advisor)

Viewing e-cigarettes more positively within cessation services was described variously by staff as a ‘shift’, ‘turnaround’ or ‘change’ and more negatively as a ‘trend’ or ‘fad’.

#### Passive approaches

All the stop smoking services in this sample were e-cigarette friendly in a passive sense. This was characterized by a tolerance of e-cigarette use by clients using their own private vaporizers/e-cigarettes within the service:

We do say our service is e-cigarette friendly so if somebody wanted to quit smoking using the e-cigarettes, we would welcome them into the service. Obviously, we won’t recommend any particular e-cigarette for them but we would provide the behavioural support for them to quit smoking (P38, commissioner)

No service reported turning away e-cigarette users or expressing disapproval of using vaping as a method of quitting. To this extent, they all fulfilled the broad definition of an e-cigarette friendly service, by accepting vaping as a personal choice of quit aid. This in itself was a recent shift for many. One service contacted about participating in early 2016 had initially declared ‘oh no, we don’t have anything to do with them (e cigarettes)’. However, by the time staff were interviewed almost 6 months later, they had all had training and were open to e-cigarette use as a matter of service policy. For some services, it had been written into their contract or ‘offer’, re-characterizing e-cigarette friendliness as a deliverable measurable target, rather than just an aspirational statement of intent. Managers and commissioners in particular, were vocal about their openness to vaping: ‘I cascaded that report (PHE) to all of Public Health… it’s really important that advisors are aware that…people can be supported to vape’ (P18, manager). This manager also acknowledged that ‘the confidence of the advisors’ was holding back the service from being truly e-cigarette friendly which they were addressing through training.

However, despite welcoming vapers who presented at the service, many services did not use the phrase ‘e-cigarette friendly’ or ‘vape friendly’ on their branding or promotional materials, such as on Twitter, in leaflets or websites. There was also concern about appearing ‘too e-cigarette friendly’. For example, one commissioner had dropped a proposal to invite e-cigarette shops to a launch event primarily because of nervousness from others in the public health team about being ‘too sort of wedded to the e-cigarette shops for want of a better way of explaining that’ (P38) as the others in the team were ‘terrified of collusion with the industry’.

#### Active approaches

A smaller number of services were more actively engaged in promoting e-cigarettes within their offer to service users. Their justification for doing so can broadly be described as motivated by social justice goals, to engage the ‘hard to reach’ groups experiencing health inequality: ‘we know that we have to do something different….We’ve got areas of the city where smoking rates are at about 30-35% and…those people, they’re not engaging with what we’re currently offering’ (P36, manager).

The need to ‘do things differently’ was particularly pressing in relation to provision for those with mental health difficulties. Many mental health/psychiatric wards were going ‘smoke-free’, having previously been exempt from public bans on smoking. For example, one city-based service did not use any e-cigarette friendly branding, but was considering e-cigarette provision for users with mental health needs:

We’re just currently coming to the end of running some focus groups for in-patients and staff around us going smoke free and how we can best support them…and e-cigarettes is the thing that’s just screaming out (P49, manager)

Only one cessation service we interviewed was currently offering e-cigarettes to service users. In 2016, this service started an e-cigarette voucher scheme in five locations including community groups (e.g. healthy living centres and voluntary organizations) in disadvantaged areas to attract unemployed, manual workers and other groups with high smoking rates. A clinic was also located within local Drug and Alcohol services to attract people engaged in substance misuse treatment. Clients were offered a combination of NRT/varenicline and an e-cigarette voucher with behavioral support, alongside other types of social provision. As one of the commissioning managers explained ‘the beauty of this offer in a community group is that they’re not just offering a prescription or a voucher, but they’re also linking the offer to the community assets that they have at hand…it might be debt management or counselling or housing…’ (P36). Working with local vaping shops was critical to the operationalization of the scheme which did not specify which product clients should use but allowed them to choose, up to the value of the voucher. Advisors noted the positive experience of working with the vape shops: ‘I think they’re just really, really professional and really caring and really genuinely want to help people quit smoking alongside me’ (P51, advisor).

Although other services were not offering e-cigarettes as an official part of their service, some had formed tentative relationships with vape shops in their area. For example, in one service, they had invited the manager of a chain of local vape stores to their staff meetings 2 years in a row to update them on vaping technology. This initiative was almost universally described positively by staff. Another service had designed a ‘Code of Conduct’ for e-cigarette shops. Another manager had tried something similar in their area and found ‘they were keen to sign up to it’; however, there were tensions over rules concerning never selling to non-smokers, as vape shop owners saw potential exemptions as justifiable (e.g. for drug harm reduction or for weight loss) (P38, commissioner). There was uncertainty, however, about what the relationship between cessation services and local vape shops could and should look like. Although overtures had been made, and many staff had visited vape shops on fact-finding visits, there were few formalized alliances or plans for longer-term interaction at this time-point, apart from the one e-cigarette voucher scheme.

### Theme two: barriers to e-cigarette integration

#### Practical barriers

Many of the barriers to using e-cigarettes within services were pragmatic ones, which were the consequence of the structure and economics of service provision, rather than any ideological resistance. Currently, e-cigarettes are not available on prescription within the NHS. Advisors were able to used prescription routes for NRT but not for e-cigarettes. Advisors, who were in touch with the everyday reality of clients’ lives, were pragmatic about the choices they were making: ‘they are people for whom change is really difficult, often they are living… in abject poverty…e-cigarettes are often a way forward but they’re too expensive… whereas nicotine replacement is on prescription and cheap’ (P14, advisor).

Many staff felt that offering e-cigarettes on prescription would be the ideal way to incorporate vaping into stop smoking services, giving it equivalence with their licensed products. However, one or two advisors were adamantly against them being on prescription: ‘no, absolutely not. They want them, they buy them…why should the NHS now start supporting their habit?’ (P13, advisor).

#### Concerns about habit and long-term use

Some staff reported they had ongoing concerns about incorporating e-cigarettes into their practice. They reported their clients had tried them and ‘they don’t get on with them’ (P11, advisor) or ‘they don’t deliver what they want’ (P14, advisor). A key issue was whether vaping broke the ‘habit’ of smoking, amid concerns it might continue their dependence: ‘it’s very easy to go back to smoking because they haven’t really broken that habit…people can vape where they couldn’t smoke before so I think they’re increasing their use’ (P11, advisor).

Some advisors were particularly concerned about the long-term use of e-cigarettes. This led them to prefer time-limited schemes similar to NRT: ‘I’d be happy to in the same way as we do nicotine replacement therapy and things like that’ (P12, advisor*).* Others saw the benefit of long-term use but were still concerned about entrenching habitual behaviour: ‘I think it’s not so much the chemical danger, it’s the behavioural danger…the door is never closed on the habit. There’s always that possibility of going back to old behaviours’ (P15, advisor).

In connection with long-term use, perhaps unexpectedly, a few advisors reported vapers approach the service about quitting vaping: ‘I have also had people coming into my clinic asking to go onto some nicotine replacement programme to come off e-cigarettes’ (P13, advisor). At least a couple of advisors were actively engaged in helping people quit vaping. This was a surprise to their manager who was emphatic they did not offer a ‘stop vaping’ service.

#### Concerns about negative health effects, safety and lack of licensed products

A few advisors were concerned about potential negative health effects of vaping: ‘e-cigarettes can cause arrhythmia and…if you quit smoking, and [have] high quantities of unregulated nicotine in an e-cigarette, it can actually cause unaccountable symptoms for a patient and that can be quite dangerous for them’ (P14, advisor). Two out of the 25 staff mentioned ‘popcorn lung’ as a genuine concern rather than in the context of media stories: ‘research has shown that…popcorn lung…is making a difference’ (P17, advisor).

More commonly, however, there were ongoing concerns about the lack of evidence relating to safety: ‘I have to explain to our patients that they are unregulated and that we cannot vouch for their safety, that there is no one product that could be used on prescription’, (P14, advisor). The lack of a prescribed or a medically ‘endorsed’ product also made many advisors nervous about suggesting them: ‘it would give me more certainty. To be put on prescription the drugs have to be inspected…passed by NICE guidelines…that would instil a bit of faith in me that what I was saying was right’ (P17, advisor). One commissioner explained the conflict of their advisors: ‘they’re not against them just for the sake of it, but they also have this duty of care and that makes them feel uncomfortable when they’re recommending products that are not either within NICE guidelines or are not medically regulated’ (P18). That said, staff were mostly aware that a medically licensed product was not likely in the foreseeable future and many argued a standardized prescribed e-cigarette would not necessarily desirable: ‘it wouldn’t really work, because they are all so diverse’ (P14, advisor).

Staff also felt relatively powerless at times in the face of ‘scare stories’ within the media:

I mean, we have got a thing on the window on our door now saying that Public Health England say they are 95% safer, and the amount of people who have no idea, they have never heard that. You know, as soon as somebody’s e-cigarette explodes, it’s in the Sun, it’s in the Mail, it’s in the Mirror’ (P39, manager)

Many felt that media scare stories were driving public attitudes towards vaping more than public health.

#### Tension over the profit/private nature of e-cigarettes supplies

A final source of concern for all staff was that public health is fundamentally a public enterprise. Engaging with a consumer product from the private sector was therefore problematic. One commissioner summed up the problem: ‘they’ve got profit in mind and we’ve got health in mind, and does that go together?’ (P37). Another commissioner found that their colleagues in public health were concerned: ‘we shouldn’t necessarily endorse any particular e-cigarette provider or any e-cigarette. I think it’s just a risk averse thing’ (P38). Even more challenging was the ‘ethical dilemma’ of potentially engaging with tobacco companies: ‘we just couldn’t get somebody turning up that, you know, from British American Tobacco or something, saying ‘oh we’ve got this vape product, but holistically, I guess you’ve got to think that people do want to stop smoking whatever the product, but it’s a difficult one. We’ll have to cross that bridge I guess’ (P18, manager). This fear of industry collusion, and of the profit motive of vape shops, was given as an explanation to account for not engaging more actively.

### Theme three: the role of public health leadership

#### Resistance from wider public health

Despite national policy changes, at a local level, there was often resistance to e-cigarettes from wider local public health. As one commissioner stated ‘I spend more time trying to convince my colleagues than doing anything else’ (P36). This often constrained local practice, for example, one service manager had tried to convince the HR (human resources) department of the council which employed them to separate their smoke-free policies on vaping and smoking in line with PHE guidance and had failed. As she stated ‘in our council policy they class vaping the same as they do smoking, despite myself and our Director of Public Health having a meeting with our HR colleague and Health and Safety to explain that actually you know, vaping could be allowed in the workplace, they chose not to’ (P37).

#### Influential figures and reports/guidance

Strong national public health leadership gave staff the confidence to challenge negative views about e-cigarettes. Particular figures and organizations within public health were mentioned throughout the interviews as the source of changing attitudes, such as key academics researching e-cigarettes, for example, ‘Robert West’ and ‘Linda Bauld’, advocacy organizations such as the ‘New Nicotine Alliance’ and other services, most notably ‘Leicester Stop Smoking Service’ which was the first e-cigarette friendly service in England:

I came back from the London one, PHE conference on e-cigarettes and you know, Peter Hajek again, he was talking, so right, that’s it, I’m going to present this to the [management board responsible for Tobacco] now…and so I’ve tried to present this very scientific evidence…I think it did start to break down some barriers… (P37, manager)

In terms of organizations, a large number of staff mentioned ‘Public Health England’ (PHE) as a key influence in giving them the confidence to engage positively around e-cigarettes:

So, as a service I’d say comparatively we were cautious to perhaps some other areas that were a little bit more, I am going to say ‘gung ho’….and then as you know Public Health England have come out and endorsed them and really been quite pro-them, and more evidence has come out then, so obviously we, on the back of that, we have been a lot more e-cigarette friendly (P49, manager)

In particular, the PHE report of 2015, containing the statistic that e-cigarettes were estimated to be 95% less harmful than combustible cigarettes, was mentioned numerous times as a key turning-point in attitude change. The report gave staff a sense of greater certainty and authority, allowing themselves to reassure others about the utility of vaping as a cessation tool: E-cigs are 95% less harmful. We are constantly saying that…to midwives for example, who are a bit reluctant to encourage people to use e-cigs (P36, advisor).

Fundamentally, participants reported, clear leadership had changed attitudes:

Originally there were [tricky conversations] because there was no clear cut what we were meant to say… it was the taboo thing you couldn’t talk about…but I now feel with all the information that’s coming out from the NCSCT about the smoking forum, the smoking pregnancy forum, it’s a lot clearer (P12, advisor)

That said, not everyone was convinced by the public health shift towards e-cigarettes. One advisor, when asked about the PHE report stated: ‘inwardly, I go no. People keep turning up at the clinics saying oh they’re safe, can you give me one…I think we need a longer period of time before we can say that, the long-term effects of them.’(P11, advisor). Another argued that public health had moved too quickly on this topic: ‘I just think they’re probably too hasty’ (P13, advisor). Others were aware that past guidance and attitudes were still influencing them:

It’s having to change our thinking, is not it? I am still in that state, you know, initially it was thought of as ‘the enemy’…’We do not recommend, we do not recommend’, I mean it’s very strong and suddenly we are saying, actually it’s ok, you know, so that’s very odd (P14, advisor)

## Discussion

Lipsky has argued that polices become practice in public service through the application of on the ground ‘discretion’ [57]. So smoking cessation services become e-cigarette friendly not only through endorsing such a policy in service descriptions, mission statements and contracts, though these are important, but through wholescale changes in practice by advisors. This occurs through what sociology terms ‘micro-social interactions’ with clients, by literally ‘being friendly’ to users of services with e-cigarettes at various points of contact that occur daily, whether on the phone, online or face-to-face. To a large extent, staff within the services sampled in this study were e-cigarette friendly in this sense. This was most notable at the managerial level [43] but many advisors were also engaged, the majority having taken part in at least one training session on e-cigarettes. In comparison with the more negative attitudes shown by health professionals in the Netherlands and USA [44, 46], the English cessation services staff in this sample were more positive about being open to working with e-cigarette users, suggesting that transitions in attitudes are occurring. However, there were limits to this positivity. There was still some use of hesitant language around ‘not being able to recommend’ e-cigarettes which the NCSCT’s guidelines suggest may be interpreted negatively by clients. Furthermore, even though all services allowed e-cigarette users to access behavioural support, most did not identify themselves in their branding and promotional literature as e-cigarette friendly. This raises the question of how potential clients would know services welcomed e-cigarettes if it was not explicitly stated. A few services were more proactive in ‘reaching out’ to vapers, for example, meeting with local vape shops or, in the case of one service offering an e-cigarette voucher for a starter kit to clinic attendees in disadvantaged areas. We conclude that there is no consensus on what constitutes an e-cigarette friendly service and that further guidelines on the specifics are needed. Nonetheless, a fundamental shift towards seeing its importance has occurred.

Additionally, this research has identified a number of important discursive themes framing service responses to vaping. One concerns the ‘morality’ or ‘ethics’ of incorporating e-cigarettes into cessation services. Both staff who were cautious and those who were enthusiastic drew on ethical and value discourses to explain their positions. The ongoing lack of a licensed e-cigarette product for prescription concerned many staff, in line with previous research [51]; this was framed here in terms of a ‘duty of care’ towards clients. There was also widespread concern about the profit motive, mirroring wider conflicts in international public health over the role of industry in driving vaping [62]. Staff more actively engaged with e-cigarette users justified their actions by drawing on ethical discourses relating to social justice and ‘care for the vulnerable’, particularly in relation to treating disadvantaged smokers or those with mental health issues. They argued ‘we have to do something different’ given the intractable nature of entrenched smoking in these groups. The battle between those who are broadly pro and anti-vaping has been characterized as one between harm reduction and prohibition [63]. This is not necessarily the case in England, where prohibitionist rhetoric is scarcer, and in which the mantra of ‘patient choice’ is more pronounced. It was notable that even the more reluctant advisors in this study did not think banning or prohibiting vaping was the way forward. Their concern was not the private use of vaping by the individual; it was about whether e-cigarettes fitted within an evidence-based, licensed and publically funded treatment programme. These concerns are not surprising, given staff are schooled in a medical model of smoking cessation treatment. We concur with Hajek that in public health ‘ideology and morality can play at least as big a role as evidence and logic’ (p. 1).

The second discursive theme identified was the importance of public health leadership and guidance. A document of key importance for this sample was the report released in 2015 by Public Health England that estimated vaping to be approximately 95% safer than tobacco smoking. Numerous staff stated that it was this statistic in particular that gave them ‘reassurance’ and ‘confidence’ to reverse their previous reluctance to engage and more openly welcome e-cigarette users into the service. This suggests that, in an ongoing climate of differing international responses, media scare stories and scientific uncertainty, public health leadership and guidance is able to perform a legitimating role for health professionals. This does not mean that many staff did not continue to have concerns, but rather that their active disengagement was reversed to an acceptance of vaping, stamped with medical authority.

There are, nonetheless, implementation issues that need to be addressed. One important contextual problem is that English stop smoking services are fragmented and constituted differently in different regions. Public Health England and the NCSCT have a primarily advisory role. Fundamentally, tobacco leads and managers are answerable to their local council boards and commissioners, who issue (or do not renew) their contracts and include (or do not include) targets relating to e-cigarettes. Our findings suggest an ongoing nervousness in wider public health and beyond about the use of e-cigarettes, evidenced by the difficulty several services reported in enacting change around working with vape shops and having separate smoking/vaping policies within council offices. As one commissioner explained, if your own council is not following clear Public Health England guidance on separating smoking and vaping for employers, your overall credibility is affected. Although many managers and commissioners in this sample were working hard to change attitudes, until this wider lack of confidence and knowledge is addressed, other public health and council colleagues (e.g. in HR) may present a significant barrier to establishing truly e-cigarette friendly services.

That said, innovative practice was occurring. In the voucher scheme example, local vape shops were chosen to receive vouchers (a redeemable method of payment from the council) so that the local population could access the intervention without having to travel. Such community-embedded initiatives may circumvent the dislike of the medicalization of e-cigarettes by some users [64] and a fear of judgment and moralization of health behaviours by health professionals [65]. It is arguable that for smoking cessation work to succeed, it is going to have to move beyond specialist clinics which few smokers attend and engage with vulnerable populations in their communities. Initiatives to support smoking cessation could occur in psychiatric units, community mental health settings, in addiction clinics, in community centres and smoke-free hospitals. E-cigarettes have the potential to be part of ‘doing things differently’ for marginalized and harder to treat smokers. For example, a Royal College of Physican’s Report in 2018 has suggested allowing vaping within hospital grounds as a smoking cessation tool [66].

It is also important to hear negative as well as positive voices concerning policy support for integrating e-cigarettes. A number of advisors with day to day experience of working with quitting smokers held negative, ambivalent or just reservations about the wholescale move towards e-cigarettes, more so than at the managerial level [43]. Their attitudes highlight experientially based issues with e-cigarettes that may explain why, although popular, up to 40% of current smokers do not want to try them, and many that have do not continue with it [67]. These include disliking being addicted to nicotine, experiential/unpleasant aspects of vaping (e.g. lung/throat sensation, feeling ‘suffocated’) and preferring medically licensed products. It is not just a matter of dismissing these as ‘myths’ about e-cigarettes, and to assert that if done correctly, vaping is pleasurable and long-term use not a problem. It may be that for some clients, using existing models of treatment such as NRT and habit-breaking [68, 69], or vaping within a more medical model of treatment with the option of a defined weaning off period, are their preferred treatment goals which align with their differential needs [64]. A ‘one size fits all’ approach may not be optimal for smoking cessation.

From a policy perspective, these results suggest several pathways forward. Services were stronger on ‘welcoming’ existing e-cigarette users than ‘reaching out’ to potential new clients as the NCSCT guidance recommends. Services should consider communicating their e-cigarette friendliness through branded/promotional material. Strong public health leadership and the sharing of successful initiatives are also important. Innovative practice is taking place in England, such as the voucher scheme discussed here. However, often managers/commissions did not know about these innovations, or if they did, were short on the details of how exactly they operated. Knowing how others have overcome concrete issues with funding, convincing others in public health and structuring interventions would be very useful. Short reports targeting service managers (as well as peer-reviewed publications) could help overcome this barrier to change. Such reports could focus on pragmatic “how-to” guidelines and provide concrete details on service implementation which are sometimes limited in journal articles.

There are several limitations to this study. Firstly, it is geographically limited to the South-West region of England. It may be that attitudes and training approaches relevant to e-cigarettes are different in the South West Public Health England network sampled here to other parts of England and elsewhere. As such, the emergent e-cigarette friendliness of this sample may not be mirrored elsewhere. Furthermore, qualitative research, whilst theoretically generative, cannot be generalized [60]. A larger-scale national study would be required to map similarities and differences, including use of quantitative methods. Finally, services were in a state of flux, meaning that the research took longer than expected to conduct. Those interviewed at the start of the data collection period may have conveyed very different views than if they had been interviewed at the end. The results presented here, like much work on e-cigarettes, may date quickly as the policy context and regulatory environment itself changes.

## Conclusions

In conclusion, given the strong association between smoking and disadvantage [70], cessation services in England have an important role in preventing further health inequalities. Even if throughput is declining within traditional stop smoking clinics, the work of smoking cessation is continuing within communities, hospitals, addiction services, psychiatric wards and in public spaces. Cessation services that are e-cigarette friendly have the potential to make considerable impact in terms of harm reduction, particularly if supported through training, policy consistency and the sharing of best practice.

## Abbreviations

ASH: Action on Smoking and Health
HR: Human resources
NCSCT: National Centre for Smoking Cessation and Training
NICE: National Institute for Health and Care Excellence
PHE: Public Health England
TPD: Tobacco Products Directive

## Footnotes

1 It is possible to vape non-nicotine products as well, although these form a small minority within the marketplace

## References

[1] Aveyard P, Arnott D, Johnson KC (2018). Should we recommend e-cigarettes to help smokers quit?. BMJ.

[2] McKee M, Capewell S. Electronic cigarettes: we need evidence, not opinions. Lancet. 2015;386. 10.1016/S0140-6736(15)00146-4. 10.1016/S0140-6736(15)00146-4 26346248

[3] Britton J. E-cigarettes, Public Health England, and common sense. Lancet. 2015;386:1238–39. 10.1016/S0140-6736(15)00145-2. 10.1016/S0140-6736(15)00145-2 26346247

[4] Stephens WE (2017). Comparing the cancer potencies of emissions from vapourised nicotine products including e-cigarettes with those of tobacco smoke. Tob Control.

[5] Sweanor D (2016). Smoking, vaping and public health: time to be creative. Can J Public Heal.

[6] Pisinger C, Døssing M (2014). A systematic review of health effects of electronic cigarettes. Prev Med (Baltim).

[7] Farsalinos KE, Stimson GV, Bell K, et al. Asking the wrong questions about e-cigarettes? A response to Stan Shatenstein. Int J Drug Policy. 2014;25:1149–50. 10.1016/j.drugpo.2014.08.001. 10.1016/j.drugpo.2014.08.001 25171909

[8] Measham F, O’Brien K, Turnbull G. “Skittles & Red Bull is my favourite flavour”: E-cigarettes, smoking, vaping and the changing landscape of nicotine consumption amongst British teenagers – implications for the normalisation debate. Drugs Educ Prev Policy. 2016;1–14. 10.1080/09687637.2016.1178708.

[9] Chatterjee K, Alzghoul B, Innabi A, et al. Is vaping a gateway to smoking: a review of the longitudinal studies. Int J Adolesc Med Health. 2016;30(3), 10.1515/ijamh-2016-0033. 10.1515/ijamh-2016-0033 27505084

[10] Tzortzi A, Teloniatis S, Matiampa G, et al. Passive exposure to e-cigarette emissions: immediate respiratory effects. Tob Prev Cessat. 2018;4 10.18332/tpc/89977. 10.18332/tpc/89977 PMC7205134 32411845

[11] Burstyn I (2014). Peering through the mist: systematic review of what the chemistry of contaminants in electronic cigarettes tells us about health risks. BMC Public Health.

[12] Kalkhoran S, Glantz SA (2016). E-cigarettes and smoking cessation in real-world and clinical settings: a systematic review and meta-analysis. Lancet Respir Med.

[13] Brown J, Michie S, Geraghty AW, et al. Internet-based intervention for smoking cessation (StopAdvisor) in people with low and high socioeconomic status: a randomised controlled trial. Lancet Respir Med. 2014;2:997–1006. 10.1016/S2213-2600(14)70195-X. 10.1016/S2213-2600(14)70195-X 25262458

[14] Hartmann-Boyce J, Begh R, Aveyard P (2018). Electronic cigarettes for smoking cessation. BMJ.

[15] Halpern SD, Harhay MO, Saulsgiver K, et al. A pragmatic trial of e-cigarettes, incentives, and drugs for smoking cessation. N Engl J Med. 2018;378:2302–10. 10.1056/NEJMsa1715757. 10.1056/NEJMsa1715757 29791259

[16] McNeill A, Brose LS, Calder R (2015). E-cigarettes: an evidence update a report commissioned by Public Health England.

[17] McNeill A, Brose LS, Calder R, et al. Evidence review of e-cigarettes and heated tobacco products 2018: a report commissioned by Public Health England. 2018. https://www.gov.uk/government/publications/e-cigarettes-and-heated-tobacco-products-evidence-review/evidence-review-of-e-cigarettes-and-heated-tobacco-products-2018-executive-summary#authors-and-citation (Accessed 7 Mar 2018).

[18] NHS Health Scotland. Consensus statement on e-cigarettes. 2017. http://www.healthscotland.scot/media/1576/e-cigarettes-consensus-statement_sep-2017.pdf (Accessed 7 Mar 2018).

[19] World Health Organization (2014). Electronic nicotine delivery systems.

[20] Schraufnagel DE, Blasi F, Drummond MB (2014). Electronic cigarettes: a position statement of the forum of international respiratory societies. Am J Respir Crit Care Med.

[21] Kennedy RD, Awopegba A, De León E (2017). Global approaches to regulating electronic cigarettes. Tob Control.

[22] Kaur J, Rinkoo AV (2015). A call for an urgent ban on E-cigarettes in India—a race against time. Glob Health Promot.

[23] Young L. New tobacco legislation to regulate sale of vaping products with nicotine nationally. Glob News. 2018; https://globalnews.ca/news/4213063/vaping-regulation-canada/

[24] Hajek P. E-cigarettes: a new foundation for evidence-based policy and practice. 2015. https://assets.publishing.service.gov.uk/government/uploads/system/uploads/attachment_data/file/454517/Ecigarettes_a_firm_foundation_for_evidence_based_policy_and_practice.pdf (accessed 29 July 2018)

[25] Bell K, Keane H. Nicotine control: e-cigarettes, smoking and addiction. Int J Drug Policy. 2012;23:242–7. 10.1016/j.drugpo.2012.01.006. 10.1016/j.drugpo.2012.01.006 22365155

[26] Moore M, McKee M, Daube M. Harm reduction and e-cigarettes: distorting the approach. J Public Health Policy. 2016;37(4):403–10. 10.1057/s41271-016-0031-2. 10.1057/s41271-016-0031-2 28202930

[27] McNeill A, Brose LS, Calder R, et al. Evidence review of e-cigarettes and heated tobacco products 2018: executive summary - GOV.UK. London: 2018. https://www.gov.uk/government/publications/e-cigarettes-and-heated-tobacco-products-evidence-review/evidence-review-of-e-cigarettes-and-heated-tobacco-products-2018-executive-summary (Accessed 21 Mar 2018).

[28] Action for Smoking on Health (2016). ASH Briefing on electronic cigarettes.

[29] Stead LF, Koilpillai P, Fanshawe TR, et al. Combined pharmacotherapy and behavioural interventions for smoking cessation. Cochrane Database Syst Rev Published Online First. 2016; 10.1002/14651858.CD008286.pub3. 10.1002/14651858.CD008286.pub3 PMC10042551 27009521

[30] McEwan A, McRobbie H (2016). Electronic cigarettes: a briefing for stop smoking services.

[31] ASH (2014). ASH Briefing: Electronic cigarettes.

[32] Tobacco Advisory Group of the Royal College of Physicians. Nicotine without smoke: Tobacco harm reduction. London: RCP; 2016. https://www.rcplondon.ac.uk/projects/outputs/nicotine-without-smoke-tobacco-harm-reduction-0 (accessed 29 July 2018)

[33] Public Health Wales. E-cigarettes/Electronic Nicotine Delivery Systems (ENDS) Position statement: PRID/S/003. 2017. www.wales.nhs.uk/sitesplus/888/opendoc/317614 (accessed 29 July 2018)

[34] National Institute for Clinical Excellence. Stop smoking support and services: NICE Guideline NG92. 2018. https://www.nice.org.uk/guidance/ng92/chapter/recommendations#advice-on-ecigarettes. Accessed 29 July 2018.

[35] Dobbie F, Hiscock R, Leonardi-Bee J (2015). Evaluating long-term outcomes of NHS Stop Smoking Services (ELONS): a prospective cohort study. Health Technol Assess (Rockv).

[36] Abraham C, Michie S (2008). A taxonomy of behavior change techniques used in interventions. Health Psychol.

[37] Michie S, Hyder N, Walia A (2011). Development of a taxonomy of behaviour change techniques used in individual behavioural support for smoking cessation. Addict Behav.

[38] Bauld L, Hiscock R, Dobbie F (2016). English stop-smoking services: one-year outcomes. Int J Environ Res Public Health.

[39] Cancer Research UK, Action on Smoking and Health. Feeling the heat: The decline of stop smoking services in England 2018. https://www.cancerresearchuk.org/sites/default/files/la_survey_report_2017.pdf. Accessed 29 July 2018.

[40] Shahab L. Integrated health behaviour (lifestyle) services: a review of the evidence. 2016. http://www.ncsct.co.uk/usr/pub/Integrated%20health%20behaviour%20services%20review.pdf (accessed 29 July 2018)

[41] Anderson W, Cheeseman H. Reading between the lines: Results of a survey of tobacco control leads in local authorities in England. A report by ASH, commissioned by Cancer Research UK. 2016. https://www.cancerresearchuk.org/sites/default/files/reading_between_the_lines_-_tobacco_control_in_england_january_2016.pdf (accessed 29 July 2018)

[42] Hiscock R, Bauld L, McEwen A. Stop Smoking Services and Health Inequalities for Smoking Cessation and Training (NCSCT) Stop Smoking Services and Health Inequalities. National Centre for Smoking Cessation and Training (NCSCT). 2013. http://www.ncsct.co.uk/usr/pub/NCSCT_briefing_effect_of_SSS_on_health_inequalities.pdf (Accessed 7 Mar 2018).

[43] Hiscock R, Bauld L, Arnott D, et al. Views from the coalface: what do English stop smoking service personnel think about e-cigarettes? Int J Environ Res Public Health. 2015;12:16157–67. 10.3390/ijerph121215048. 10.3390/ijerph121215048 PMC4690984 26703638

[44] Van Gucht D, Baeyens F. Health professionals in Flanders perceive the potential health risks of vaping as lower than those of smoking but do not recommend using e-cigarettes to their smoking patients. Harm Reduct J. 2016;13 10.1186/s12954-016-0111-4. 10.1186/s12954-016-0111-4 PMC4919883 27342543

[45] Ofei-Dodoo S, Kellerman R, Nilsen K (2017). Family physicians’ perceptions of electronic cigarettes in tobacco use counseling. J Am Board Fam Med.

[46] Cummins S, Leischow S, Bailey L (2016). Knowledge and beliefs about electronic cigarettes among quitline cessation staff. Addict Behav.

[47] Kollath-Cattano C, Thrasher JF, Osman A (2016). Physician advice for e-cigarette use. J Am Board Fam Med.

[48] Singh B, Hrywna M, Wackowski OA (2017). Knowledge, recommendation, and beliefs of e-cigarettes among physicians involved in tobacco cessation: a qualitative study. Prev Med Reports.

[49] Egnot E, Jordan K, Elliott J (2017). Associations with resident physicians’ early adoption of electronic cigarettes for smoking cessation. Postgrad Med J.

[50] Hiscock R, Goniewicz ML, McEwen A, et al. E-cigarettes: online survey of UK smoking cessation practitioners. Tob Induc Dis. 2014;12:13. 10.1186/1617-9625-12-13. 10.1186/1617-9625-12-13 PMC4147097 25170337

[51] Beard E, Brose LS, Brown J, et al. How are the English stop smoking services responding to growth in use of electronic cigarettes? Patient Educ Couns. 2014;94:276–81. 10.1016/j.pec.2013.10.022. 10.1016/j.pec.2013.10.022 24290243

[52] Tamimi N. Knowledge, attitudes and beliefs towards e-cigarettes among e-cigarette users and stop smoking advisors in South East England: a qualitative study. Prim Health Care Res Dev. 2017:1–8. 10.1017/S1463423617000445. 10.1017/S1463423617000445 PMC6452951 28774353

[53] Sherratt FC, Newson L, Marcus MW (2016). Perceptions towards electronic cigarettes for smoking cessation among Stop Smoking Service users. Br J Health Psychol.

[54] Sherratt FC, Marcus MW, Robinson J, et al. Electronic cigarette use and risk perception in a Stop Smoking Service in England. Addict Res Theory. 2015;23:336–42. 10.3109/16066359.2015.1006629.

[55] Rooke C, Cunningham-Burley S, Amos A. Smokers’ and ex-smokers’ understanding of electronic cigarettes: a qualitative study. Tob Control. 2015;25 (issue e1), 10.1136/tobaccocontrol-2014-052151. 10.1136/tobaccocontrol-2014-052151 26055267

[56] Hajek P, Corbin L, Ladmore D, et al. Adding e-cigarettes to specialist stop-smoking treatment: City of London Pilot Project. J Addict Res Ther. 2015;6:244. 10.4172/2155-6105.1000244.

[57] Lipsky M. Street-level bureaucracy: Dilemmas of the individual in public service. New York: Russel Sage Foundation; 1980.

[58] Bell K, Stimson GV (2015). Nicotine: science, regulation and policy. Int J Drug Policy.

[59] Stimson GV, Thom B, Costall P. Disruptive innovations: the rise of the electronic cigarette. Int J Drug Policy. 2014;25:653–5. 10.1016/j.drugpo.2014.05.003. 10.1016/j.drugpo.2014.05.003 24889671

[60] Patton MQ. Qualitative research & evaluation methods: Integrating theory and practice. 4th ed. Thousand Oaks: Sage Publications Ltd; 2014.

[61] Braun V, Clarke V (2006). Using thematic analysis in psychology. Qual Res Psychol.

[62] Fairchild AL, Bayer R. Public health. Smoke and fire over e-cigarettes. Science. 2015;347:375–376. doi:10.1126/science.1260761. 10.1126/science.1260761 25613878

[63] Hajek P. Electronic cigarettes have a potential for huge public health benefit. BMC Med. 2014;12:225. 10.1186/s12916-014-0225-z. 10.1186/s12916-014-0225-z PMC4260378 25491742

[64] Farrimond H (2017). A typology of vaping: identifying differing beliefs, motivations for use, identity and political interest amongst e-cigarette users. Int J Drug Policy.

[65] Jakobsen SP, Charlotte Overgaard C (2018). ‘They’ll be judging us’ a qualitative study of pregnant women’s experience of being offered participation in a supportive intervention. Midwifery.

[66] Tobacco Advisory Group of the Royal College of Physicians. Hiding in plain sight: treating tobacco dependency in the NHS. London: RCP; 2018. https://www.rcplondon.ac.uk/projects/outputs/hiding-plain-sight-treating-tobacco-dependency-nhs (accessed 29 July 2018)

[67] Simonavicius E, McNeill A, Arnott D, et al. What factors are associated with current smokers using or stopping e-cigarette use? Drug Alcohol Depend. 2017;173:139–43. 10.1016/J.DRUGALCDEP.2017.01.002. 10.1016/j.drugalcdep.2017.01.002 PMC5380653 28246049

[68] West R, McNeill A, Raw M (2000). Smoking cessation guidelines for health professionals: an update. Health Education Authority. Thorax.

[69] Dean J (2013). Making habits, breaking habits.

[70] Jarvis M, Wardle J, Marmot M, Wilkinson RG (1999). Social patterning of individual health behaviours: the case of cigarette smoking. Social determinants of health.

